# A community based intervention program to enhance neighborhood cohesion: The Learning Families Project in Hong Kong

**DOI:** 10.1371/journal.pone.0182722

**Published:** 2017-08-21

**Authors:** Chen Shen, Alice Wan, Lit Tung Kwok, Sally Pang, Xin Wang, Sunita M. Stewart, Tai Hing Lam, Sophia S. Chan

**Affiliations:** 1 School of Public Health, Li Ka Shing Faculty of Medicine, The University of Hong Kong, Hong Kong SAR, China; 2 Christian Family Service Center, Kwun Tong, Hong Kong SAR, China; 3 Department of Psychiatry, The University of Texas Southwestern Medical Center at Dallas, Dallas, TX, United States of America; 4 School of Nursing, Li Ka Shing Faculty of Medicine, The University of Hong Kong, Hong Kong SAR, China; Institut Català de Paleoecologia Humana i Evolució Social (IPHES), SPAIN

## Abstract

**Background:**

Neighborhood cohesion, which refers to the extent of the connectedness and solidarity among residents in a community or neighborhood, is an important determinant of human health. To enhance neighborhood cohesion, the “Learning Families Project” was developed with a series of intervention programs in Kwun Tong in Hong Kong, a district with low neighborhood cohesion. This project, based on the social ecological model, provided a platform for neighbors to learn, communicate and interact with each other.

**Methods:**

This quasi-experimental study included two nearby government subsidized low rent housing estates separated by busy main roads. One served as the intervention (Tsui Ping (South) Estate) and one as the control (Shun Tin Estate) estate. The intervention included promotion, resident training and learning programs, embodied by a series of community activities such as talks, day camp, thematic activities and horticulture class. Baseline (before the programs) and follow-up (one year after the programs) surveys were conducted both in the intervention and control estate to assess the impact of the programs on neighborhood cohesion.

**Results:**

The number of residents who completed both the baseline and follow-up surveys was 502 in the intervention estate and 476 in the control estate. Neighborhood cohesion significantly improved in the intervention group after the programs (Cohen effect size d: 0.15). Compared with the control group, the improvements in closeness of the neighborhood and trust in neighbors were significantly greater in the intervention group (Cohen effect size d: 0.13 and 0.14, respectively).

**Conclusion:**

This brief intervention program using a quasi-experimental study design increased neighborhood cohesion in a low rent housing estate.

**Trial registration:**

ClinicalTrials.gov NCT02851667

## Introduction

Neighborhood is a geographically localized place where residents interact face-to-face with each other to realize common values, socialize youth and maintain effective social control [1]. Neighborhood cohesion refers to the extent of the connectedness and solidarity among residents in a community or neighborhood [2]. Neighborhood cohesion includes many domains, such as common values, trust, as well as mutual aids and support [3]. Neighborhood cohesion, a collective characteristic of communities, is associated with better perceived physical and mental health [4–6] as well as lower risks of stroke [7], functional limitations [8] and depression [9]. Possible mechanisms might be that neighborhood cohesion is associated with healthier behaviors such as less smoking and more physical activity [10–12]. In addition, trust in neighbors is also protective against stress, which might be a plausible mechanism linking trust and mental health [13]. Although the benefits of neighborhood cohesion are increasingly recognized, there are few reports in the literature of effective strategies at the community level to enhance solidarity and trust among residents of a community.

Kwun Tong is a district in Hong Kong with relatively low neighborhood cohesion, based on a territory-wide household survey [14]. Only 38.7% of the participants reported that they could trust their neighbors, and 37.0% of the participants reported that their neighborhood was close-knit. The low socio-economic status of Kwun Tong residents, reflected in low education attainment and household income[15], possibly explains the low neighborhood cohesion [16]. In addition, the prevalence of family problems such as elderly abuse, domestic violence and child abuse is also high in Kwun Tong [17, 18]. The “Learning Families Project” (LFP) consisted of a series of community-based intervention programs designed to enhance family well-being and neighborhood cohesion in Kwun Tong. The LFP was part of the project entitled ‘FAMILY: a Jockey Club Initiative for a Harmonious Society’ (the FAMILY project) that included a longitudinal family cohort study [19], other intervention studies [20–23], and social marketing programs [24]. The FAMILY project focused on the family as a unit and aimed to identify the sources of family problems, devise appropriate preventive measures, and promote family 3Hs.

The LFP was initiated based on the social ecological model. This social ecological model proposes dynamic interrelations among various personal and environmental factors [25]. The model emphasizes that people’s behaviors are affected by intra-personal, inter-personal, community and societal factors [26]. LFP promoted the concepts of Learning Family and family health, happiness and harmony (family 3Hs) to the participants (intra- and inter personal level), and was innovative in extending across multiple levels of factors. The concept of “Learning Family” indicates that family communication and family well-being can be promoted when family members participate in learning activities together [27]. Family 3Hs are conceptualized as the main themes of family well-being in Chinese settings [28]. Family health includes physical and mental health of family members, which are strongly related to psychological capital and family unity [28]. Family happiness can be enhanced by spending time with family members and building connection with friends and relatives. Family harmony means absence of conflicts and effective communication with family members. Forbearance and spending time with family are important in forming a harmonious family [28]. LFP provided a platform for family members to spend time together and communicate with each other through learning something useful and interesting (inter-personal level), as well as for neighbors to interact and build social networks while learning together in and after these community activities (community level). Other community-based projects that have focused on collective participation have shown improvement in social cohesion [29, 30].

Previous intervention programs using social ecological models have required intensive involvement from both the service providers and recipients [31, 32], perhaps making such kind of programs difficult to sustain and disseminate. Our previous brief community based intervention programs, often with a core session and booster session, yielded some small but significant improvement on family communication, parent-child relationships, and family well-being [23, 33]. The present paper aimed to examine whether community based intervention programs with modest intensity could extend the effect to the community and enhance neighborhood cohesion.

## Methods

### Participants

Participants were recruited by a large charitable non-governmental social welfare organization, Christian Family Service Center (CFSC), with the mission to support and enhance family functioning and to foster an environment for growth and change in this district. CFSC provides an array of services such as child and family services, youth services, and elderly care services [34]. CFSC collaborated with School of Public Health, The University of Hong Kong (HKU) in planning, and implementing the LFP.

We chose Tsui Ping (South) Estate (about 17,000 residents in 2011) [35] as the intervention site, and Shun Tin Estate (about 19,000 residents in 2011) [36] as the control site. As the two estates offer government subsidized, public, low rent housing, the residents are of similar socio-economic backgrounds. They were located about 2.5 km apart, and are well separated by busy main roads, minimizing the likelihood of cross-social relationships and transfer. Residents living in the designated estates were eligible if they were Hong Kong residents, older than 10 years of age, and could communicate in Chinese (Cantonese or Putonghua). We utilized a convenience sample. The headquarter of CFSC is located at a few minutes’ walk from Tsui Ping (South) Estate.

**Fig 1:**
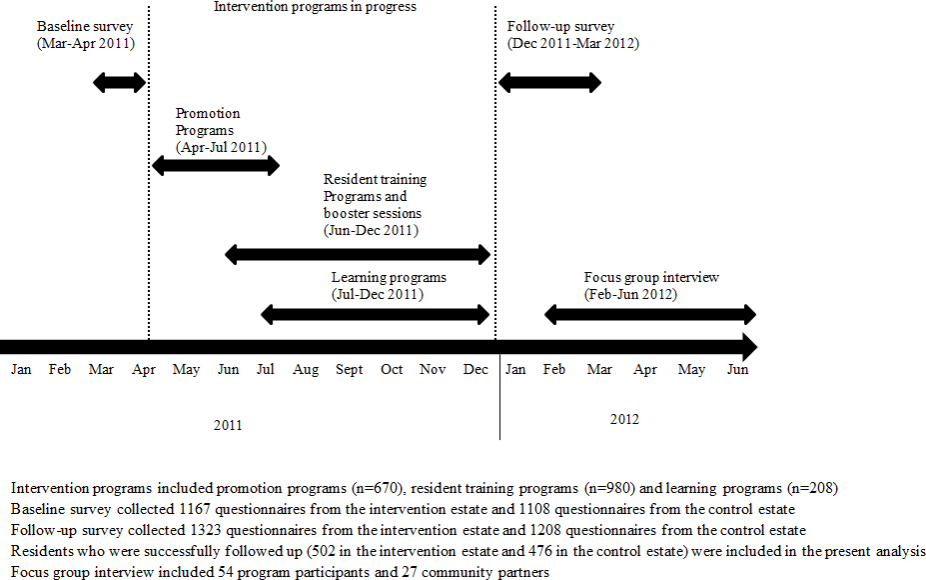
Timeline for learning families project.

Key community stakeholders such as the Estate Management Advisory Committees (EMAC) and the Mutual Aid Committees (MAC) in the intervention estate were actively engaged as key partners in this study, including joining focus group interviews at the needs assessment stage. The assessment explored their views on family well-being and neighborhood cohesion as well as resources and feasibility of this study. A train-the-trainer program was designed and implemented from December 2010 to February 2011 to engage and equip resident leaders from both EMAC and MAC to recruit participants and organize family programs with the Learning Family concepts and leadership skills. Fig 1 shows the timeline of LFP.

### Fieldwork recruitment

Fieldwork recruitment of residents took place from March to December 2011. A diverse array of recruitment strategies were used concurrently, including posters, leaflets, and banners, a kick-off ceremony, promotion activities, telephone calls, mobile counter, and door-to-door canvassing to raise awareness of the LFP in the community residents. Resident leaders, CFSC project staff, and academic staff, were actively involved in the recruitment process.

#### Posters, leaflets and banner

Approximately 19,200 leaflets were distributed into mailboxes of the intervention estate, recreational centers, local and community organizations, and at community events at various time during recruitment. In addition, 688 posters were displayed in various community organizations, housing blocks, community events, Estate Management Company, and Housing Authority office. One banner was also displayed in a conspicuous area in the estate. Community organizations and individuals were encouraged to forward and disseminate information to others who might be interested.

#### Kick-off ceremony

A kick-off ceremony was launched in March 2011 at a basketball court in the estate to promote a sense of project ownership in community partners as well as to increase visibility of the project and the research team to the community residents. Residents were notified of the event via posters, word of mouth, and a banner set up in the estate. All of the members of the steering committee attended along with representative from the funding source (Executive Director of The Hong Kong Jockey Club Charities), Housing Authority (Deputy Director), District Council (Chairman), Social Welfare Department (Assistant Director of Family and Child Welfare), and a Hong Kong Television celebrity. Stage performances and game booths were hosted by CFSC project staff, resident leaders, and volunteers (most of which were local residents). Logo-adorned items that carried the project name and leaflets with information about the LFP were also distributed.

#### Promotion programs

A total of 10 promotional and publicity activities, such as “Easter egg painting activity” and “DIY photo frames as Mother’s Day gift” commenced in the intervention estate by the CFSC from April to July 2011 to raise awareness with regards to the concepts of “Learning Family” and “Family 3Hs” as well as help recruit members into the LFP and disseminate information about the upcoming resident training programs and learning programs. The duration of each program was less than three hours. A total of 670 residents joined the promotion programs. Of these, 293 enrolled as members of LFP and left their contact specifics with the CFSC so as to receive updated information about the project.

#### Telephone calls

Active outreach was conducted by direct contact of potential participants by CFSC staff. Approximately 1000 telephone calls were made to potential participants. Word of mouth was also utilized by CFSC staff to contact community organizations to spread the recruitment message. Similarly, word of mouth referrals were used by resident leaders and volunteers to recruit residents. Residents who participated were also encouraged to share and inform others who might be interested, serving as agents to expand recruitment.

#### Mobile counters

Fifty-one mobile counters were set up in the estate at various times during recruitment. To increase residents’ interest, a free health check was set up and managed by nursing students from HKU, the academic partner. Staff made face-to-face contact with passerby residents to distribute leaflets, introduce the project and recruit residents into the LFP. These mobile counters similar to the other promotion activities gave community residents information on the aims of the project and personal contact of the researchers and project staff. Daytime and evening, weekday and weekend slots were necessary to access a cross-section of the estate residents.

#### Door-to-door canvassing

To reach the hard-to-reach residents, door-to-door visits were conducted. A total of 10 visits reaching approximately 1000 households were made at various times. For each visit, a total of four staff members including CFSC project staff, resident leaders, and volunteers approached the residents by knocking on the door of each apartment unit during weeknights and weekends. The team introduced the project aims and invited residents to participate. Logo-adorned items (e.g., chopsticks, shopping bags) that carried the study name were distributed as incentives to increase interest.

### Program development

#### Resident training programs

Resident training programs were the main intervention of LFP. Based on the information from the needs assessment, 24 resident training programs such as talks, day camp and thematic activities were delivered in the intervention estate by CFSC from June to November 2011. Each program included an introduction to the concepts of Learning Family and family 3Hs as well as how to promote family 3Hs through family learning, delivered by interactive games and workshops. The workshops included cooking, handwork and exercise enabling participants to learn and communicate with each other. Resident training programs except for day camp were held in CFSC headquarters with the duration limited to two hours to enhance recruitment and reduce costs. The day camp was held in a holiday camp and the duration of content related to Learning Family was limited to two hours. Participants completed a questionnaire before the above activities (T1) and immediately afterwards (T2). A 26-page booklet (Learning Family Booklet), produced as a tool for training and also used as a record book for the participants to document their participation in the learning activities as well as their learning contents, was distributed to each participant. The number of participants in the resident training programs was 980, with 515 valid questionnaires at T1 and 444 valid questionnaires at T2.

**Fig 2:**
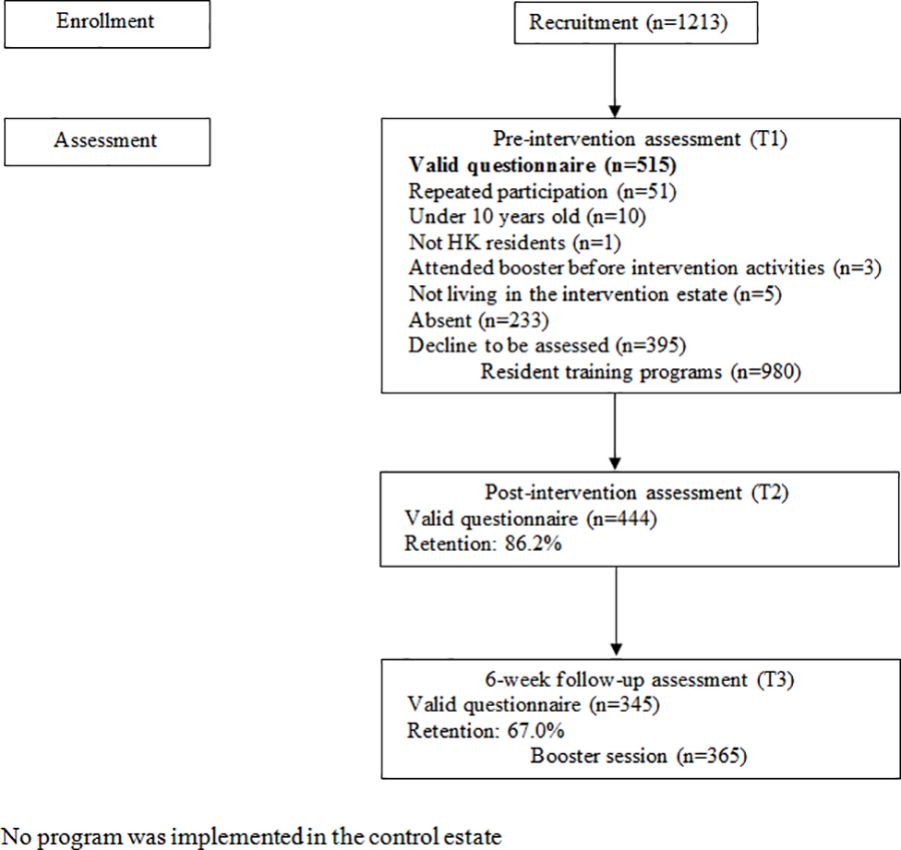
The flow diagram of participants in the intervention group.

A total of six booster sessions were held six weeks after the resident training programs for the participants in the New Life Interactive Farm by CFSC from July to December 2011. The duration of each session was three hours. Each participant in the booster session had attended one resident training program and had completed a valid questionnaire at T1. The participants had a guided tour of the farm and two experiential activities (organic farming and seed learning). Debriefing and reviews of the concepts of Learning Family and family 3Hs were also provided. Questionnaire survey was conducted after the booster activities (T3). The number of participants in the booster sessions was 365, with 345 valid questionnaires at T3. Each participant with a valid questionnaire at T3 received one resident training program and one booster session. The flow diagram of resident training programs is presented in Fig 2.

#### Learning programs

Learning programs were conducted in the intervention estate by the MACs members in each housing blocks from July to December 2011 to further consolidate the concept of Learning Family and family 3Hs. A total of 14 learning programs were delivered as classes on topics designed to be of broad interest such as laughing yoga, craft making, healthy eating and exercise classes. Some classes had multiple sessions such as a horticulture class (four sessions). Each class also contained an introduction of the concepts of Learning Family and family 3Hs. Residents in the intervention estate were encouraged to learn something new with their family members. These activities were designed to be enjoyable and suitable for people of all ages. Participants could enjoy each other’s company in a supportive environment during the activities. Learning programs were conducted in CFSC headquarters or the community basketball court. The duration of each session of each program ranged from one to two hours. The Learning Family Booklet was also distributed to each participant. The number of participants in the learning programs was 208.

### Baseline and follow-up survey

We conducted a baseline (before the intervention programs) and a follow-up survey (one year after the intervention programs) in the intervention and control estates using a self-administrated questionnaire in order to evaluate the whole impact of the programs on the community. The questionnaires were delivered and collected by multiple methods such as mobile counters, security desk in housing blocks, door-to-door visits and street booths.

A total of 1167 and 1108 residents in the intervention and control estates respectively participated in the baseline survey, and 1323 and 1108 residents in the intervention and control estates respectively participated in the follow-up survey. A total of 502 and 476 residents from the intervention and control estate respectively were successfully followed up (the same person completed both the baseline and follow-up questionnaires) using record linkage based on the name and residential address, and were included in the present analysis. Among 502 residents from the intervention estate, 30.7% of them (n=154) had participated in at least one of the intervention programs. The baseline survey was conducted from March 2011 to April 2011, and the follow-up survey was conducted from December 2011 to March 2012.

### Focus group interview

Six focus group interviews were conducted with 54 participants who had participated in the resident training programs to explore their experiences in the programs, their mastery of Learning Family concepts, as well as the changes in their family 3Hs and neighborhood cohesion. The number of participants in each focus group ranged from six to 12.

Five focus group interviews were also conducted with 27 community partners (three for EMAC/MAC, one for CFSC staff and one for peer counsellors) after the programs to explore their experiences with the project including perceptions of challenges, personal growth and sharing of experiences with others. The number of participants in each focus group ranged from three to eight.

These interviews were conducted from February to May 2012 in a quiet venue (e.g. an activity room) and lasted about 60 minutes. Each group was managed by a panel of three members, which consisted of one moderator and two note-takers.

### Outcomes

Neighborhood cohesion was measured using a five-item neighborhood cohesion scale [37]. Participants were asked how strongly they agreed that “people around here are willing to help their neighbors”, “this is a close-knit neighborhood”, “people in this neighborhood can be trusted”, “people in this neighborhood generally do not get along with each other”, “people in this neighborhood do not share the same values”. Response choices included “strongly disagree”, “disagree”, “neutral”, “agree”, and “strongly agree”, scored from one to five (the last two statements were reverse coded). The scale was translated into Chinese. A total neighborhood social cohesion score was calculated, with a higher score indicating greater neighborhood cohesion. In this study, the Cronbach’s α coefficient ranged from 0.93 to 0.95 for the scale at baseline and follow-up surveys in both estates, indicating good internal consistency [38].

### Fidelity check

Fidelity of the interventions was monitored by HKU academics and CFSC staff. It included program quality and program objectives achievement, both of which were rated on a single item for adherence to program content, with a score scale ranging from 0 to 100. Fidelity checks showed that the mean scores of both program quality and program objectives achievement were more than 75, indicating that the interventions were delivered to participants successfully as planned.

### Ethical statement

Ethical approval was granted by the Institutional Review Board (IRB) of the University of Hong Kong / Hospital Authority Hong Kong West Cluster. Written informed consent was obtained from all participants before the start of the programs. For participants younger than 15, the consent was signed by their parents or legal guardians on behalf of them. This study was registered under ClinicalTrials.gov (NCT02851667).

### Statistical analysis

Chi-square tests and analysis of variance (ANOVA) were used to compare baseline characteristics between participants in the intervention and control groups. Paired t-test was used to assess the mean change of score in the neighborhood cohesion scale in the intervention and control groups. A multivariate multi-level analysis was used to assess the difference of mean change in the neighborhood cohesion scale between the intervention and control groups. We controlled for potential confounders specifically age and education level. Correlations among individuals from the same family, and cluster effect of individuals living in the same block were modeled as random effects with different variance. Effect size was measured using Cohen’s d. A Cohen’s d of 0.20 was described as a small effect, 0.50 as a medium effect, and 0.80 or above as a large effect [39].

Qualitative data were analyzed by a panel of two researchers independently; one attended the focus group interviews, while the other was absent, an arrangement promoting accuracy as well as objectivity during analysis. The strategy of thematic content analysis was used [40]. Each transcript was analyzed sentence by sentence and coded for respondents’ meanings. Initial open coding of the data used differing codes, which were then organized into categories and themes. Consensus was reached through discussion and iterative review of codes, categories and themes by the two researchers. All codes were then reviewed together by the research team to minimize the likelihood of their own biases and viewpoints embodied in the themes.

We began by familiarizing ourselves with the data and utilized a process of “constant comparison” during analysis. This essentially involved reading and re-reading the data to search for and identify emerging themes. This process began in the initial coding stage when the researchers began to reflect on the data, broke down the content into discrete parts, and examined each quote for similarities and differences. For instance, we identified quotes such as “communicate and get to know the neighbors”, “enjoy activities with the neighbors”, or “greet the neighbors” as similar content that reflected the improvement of neighborhood cohesion through communication. Open coding was conducted at the first stage, and initial codes were developed. The researcher collected all the initial codes together and worked through to identify any duplication, overlap, or similar categories. Some of these codes were further refined and reduced in number by grouping them together. For example, our initial codes included “(after the program) some of the elderly residents would greet us”, “they (the participants) know more neighbors after joining the activities” and other similar codes that were later organized into a broad category of “positive impact on neighborhood relationships”. Through the use of sentence by sentence coding, we were able to further organize these codes into broad categories, themes, and subthemes when appropriate. For instance, the codes “the participants made friends with their neighbors”, and “neighbors had more topics to chat about (following participation)” were categorized into the broad theme of “program effectiveness”. These were then further organized into the sub-theme “improvement in neighborhood cohesion”.

## Results

Table 1 shows that age, sex, education attainment, and monthly household income of the participants were similar in the intervention and control groups, but neighborhood cohesion was lower in the former.

**Table 1:** Baseline characteristics between the intervention and control group.

Characteristics	Intervention (n=502)	Control (n=476)	P-values^a^
Sex (%)			
Men	30.0	29.8	
Women	70.0	70.2	0.93
Age (%)			
<18	1.0	3.4	
18-44	35.7	33.9	
45-64	30.1	32.4	
65+	33.1	30.3	0.06
Education level (%)			
No formal education	17.7	14.0	
Primary	25.0	28.8	
Secondary or above	57.3	57.2	0.18
Monthly household income (%)			
<HK$4000 (US$1=HK$7.8)	24.4	18.6	
4000-7999	19.7	18.8	
8000-9999	13.2	18.0	
10,000-14,999	20.9	20.6	
≥15,000	21.8	24.0	0.10
Neighborhood cohesion scale^b^	3.26 (0.57)	3.36 (0.57)	<0.001

a: P values for two-sided chi-square test for demographic characteristics and ANOVA for neighborhood cohesion

b: Mean (standard deviation)

Table 2 shows that neighborhood cohesion significantly improved with a small effect size (Cohen effect d: 0.15) in the intervention group, but did not change in the control group. Closeness, trust and value uniformity of the neighborhood also improved significantly with a small effect size (Cohen effect d: 0.15, 0.11 and 0.23, respectively) in the intervention group and uniformity also improved in the control group (Cohen effect d: 0.34).

**Table 2:** Mean change of scores in the neighborhood cohesion before and one year after the programs.

	Estate	Baseline (mean (SD))	Follow-up (mean (SD))	Effect size^a^
Neighborhood cohesion scale^b^	Intervention	3.26 (0.57)	3.34 (0.66)	0.15*
	Control	3.36 (0.57)	3.39 (0.62)	0.07
Item 1: People around here are willing to help their neighbors	Intervention	3.43 (0.86)	3.48 (0.84)	0.08
	Control	3.58 (0.80)	3.53 (0.80)	0.08
Item 2: This is a close-knit neighborhood	Intervention	3.30 (0.88)	3.39 (0.86)	0.15*
	Control	3.42 (0.85)	3.41 (0.85)	0.03
Item 3: People in this neighborhood can be trusted	Intervention	3.36 (0.81)	3.43 (0.81)	0.11*
	Control	3.53 (0.75)	3.46 (0.78)	0.11
Item 4: People in this neighborhood generally do not get along with each other	Intervention	3.35 (0.76)	3.33 (0.87)	0.03
	Control	3.38 (0.86)	3.42 (0.83)	0.06
Item 5: People in this neighborhood do not share the same values	Intervention	2.89 (0.74)	3.07 (0.86)	0.23**
	Control	2.90 (0.77)	3.15 (0.85)	0.34**

Intervention group (n=502); Control group (n=476)

a: Cohen effect size d: small=0.20, medium=0.50, and large=0.80

b: Scores for each item ranged from 1 to 5, with higher scores indicating better outcomes. Scores in the neighborhood cohesion scale were the average score of items (Items 4 and 5 were reverse coded)

*: Statistically significant at P<0.05;

**: Statistically significant at P<0.001;

Table 3 shows that compared with the control group, neighborhood cohesion in the intervention group improved more, although the increase was not significant. However, the improvement of closeness and trust of neighborhood in the intervention group was significantly greater, with a small effect size (Cohen effect d: 0.13 and 0.14, respectively). Relevant details of the multivariate multi-level analysis including the coefficients at individual, family and block levels and the fraction of total variance due to the variation at the family and block levels were shown in S1 Table.

**Table 3:** Differences in changes of scores in neighborhood cohesion scale in the intervention and control group.

	Mean change^a^		P value	Effect size^b^
	Intervention	Control		
Neighborhood cohesion scale^c^	0.08	0.03	0.32	0.07
Item 1: People around here are willing to help their neighbors	0.06	-0.05	0.09	0.11
Item 2: This is a close-knit neighborhood	0.10	-0.02	**0.03**	0.13
Item 3: People in this neighborhood can be trusted	0.08	-0.07	**0.02**	0.14
Item 4: People in this neighborhood generally do not get along with each other	-0.02	0.05	0.30	0.07
Item 5: People in this neighborhood do not share the same values	0.18	0.26	0.25	0.07

Intervention group (n=502); Control group (n=476)

a: Change from pre-program to one year after the programs

b: Cohen effect size d: small=0.20, medium=0.50, and large=0.80

c: Positive changes in scores indicated improved outcomes. Scores in the neighborhood cohesion scale were the average score of items (Items 4 and 5 were reverse coded)

Three themes emerged from the participants’ focus groups. “Program effectiveness” consisted of the implementation of the concepts of Learning Family, improvement in family communication, family relationship and family 3Hs, improvement in neighborhood cohesion, and challenges encountered. “Participants’ perception about involvement of the members of EMAC and MACs” consisted of their efforts in promoting the activities and assisting the logistics arrangement, as well as their roles and contributions in this project. “Participants’ overall comments and recommendation for the project” consisted of the content of the programs and the questionnaires for assessment.

When asked about their opinions on this project, the participants indicated that this kind of project was worthwhile to continue. They welcomed the activities that focused on improving family relationships. Furthermore, they recommended that activities could be tailored to the needs of different age groups.

*“You can design some games for kids*. *For the elderly*, *gymnastic exercises or other activities are also good for them*.*”* (A mother, Group 6, 34A)

The programs had some effects on the community as neighborhood relationships improved. The participants made friends with their neighbors and joined some activities together. They had more topics to chat about. Some even showed more caring behaviors to their neighbors.

*“(The program) gave me a chance to communicate and get to know the neighbors*. *I can also enjoy activities with the neighbors*, *in addition to my family*.*”* (A mother, Group 5, 383C)

*“I greeted the neighbors*. *Sometimes*, *when I met other housewives*, *I asked ‘Are you looking for work*?*’ When I knew there was a job vacancy in the factory*, *I helped make the connection*. *She could work there if she was interested*.*”* (A mother, Group 3, 37A)

Four themes emerged from the community partners’ focus groups. “Program effectiveness from the perspectives of community partners” consisted of the enhancement of family 3Hs, social networking, and neighborhood cohesion of the community from the point of view of community partners. “Roles of community partners” consisted of promoting and recruiting participants, running game booths, and explaining the questionnaires to the participants. “Challenges and barriers encountered” consisted of the issue of questionnaire characteristics (long, repetitive and difficult to understand) and the difficulty of motivating the residents to participate. “Suggestions on future project implementation” consisted of allocation of resources, more effective communication and partnership, and involvement and cooperation of local community parties such as District Council members.

The community partners also agreed that the project had a positive impact on the community and there were noticeable changes in neighborhood relationships.

*“The change is great*. *(After the program) some of the elderly residents*, *who usually sit and rest downstairs in the morning or at night*, *would greet us now*.*”* (A MAC member, Group 1, 16)

*“They (the participants) have gotten to know more neighbors after joining the activities*.*”* (A CFSC staff, Group 4, 1)

## Discussion

Our findings show that neighborhood cohesion increased after our community based intervention programs. Compared with the control group, the intervention group showed slightly more increase in neighborhood cohesion, and increases in the closeness of neighborhood and the trust in neighbors were significantly greater. The quantitative measures were corroborated by the qualitative assessments which helped evaluate the outcomes more comprehensively.

By the involvement of community residents in the intervention programs with an emphasis on family communication and family activities, the present study shows that the intervention based on the social ecological model can enhance neighborhood cohesion. These findings suggest that the social ecological model, which has typically been utilized to guide changes in individuals’ health behaviors[41], can be modified and extended to benefit the community.

Although the difference in improvement in neighborhood cohesion between the intervention and control group was not significant, within group improvement in the intervention group was observed after the programs. It is possible that the low intervention intensity may be insufficient to produce a detectable between-group effect. Previous intervention programs using social ecological model often contained multiple intensive sessions [31, 32]. As the main intervention of LFP, our resident training programs were very brief with only one core session and one booster session (total five hours). As expected, primary prevention interventions have modest effects probably because of the low intensity of the intervention. However, these interventions are most useful when they can be disseminated to large numbers of people. From the public health point of view, brief interventions are expected to attract more participants and enhance the feasibility and retention rate, as well as reduce the cost of training the interventionists and delivering the programs. There may also be a cultural reason for the small effect size because Chinese people may be more “cautious” because moderation is favored in Chinese culture[42]. Very small number of participants chose “strongly disagree” or “strongly agree” with these statements on neighborhood cohesion. The general reluctance of Chinese to choose the most extreme answer options might result in difficulties detecting moderate effect size based on the five-point scale.

Nevertheless, the increases in the closeness of neighborhood and trust in neighbors were significant. These community activities and workshops provided a physical location for residents to meet each other, socialize, and learn some skills in their local community [29, 30]. Other elements did not change substantially perhaps because the programs did not focus on these aspects. Our study adds to the literature that our brief community based programs could benefit the community and improve neighborhood cohesion.

The collaboration of academics and the social service sector enabled the successful delivery of this community-based intervention program. However, the delivery of such collaborative interventions might not be a routine practice in other social contexts. Our findings suggest that academic-community collaborations should be considered in other social contexts.

Our study has some limitations. First, although we chose two well-separated estates, we could not rule out contamination. Second, the participants were not randomized individually, and neighborhood cohesion in the intervention group at baseline was lower than the control group. Third, not all the participants in the surveys had participated in LFP, which might lead to the reduced effect size. Fourth, there could be a ceiling effect as some participants came with already high scores in neighborhood cohesion before the programs. Fifth, no efforts were made to engage the control community in learning or activities to serve as a “placebo” control. Sixth, the neighborhood cohesion scale has not been formally validated in Hong Kong.

There is a Chinese saying that “Distant kinsmen mean less than close neighbors”, acknowledging that even this family-oriented culture, physical community is important. As these intervention programs mainly focused on family rather than neighborhood, further studies on the effectiveness of similar public health programs on neighborhood cohesion in Chinese community are warranted. Scales that take into account the tendency to use a restricted range would be valuable.

## Conclusion

Hong Kong is the most westernized, urbanized and economically developed city in China, with residents sharing cultural values including those emphasizing relationships. Our brief community-based intervention programs based on the social ecological model improved indices of neighborhood cohesion, specifically the closeness of neighborhood and trust in neighbors in a low rent housing estate where residents have relatively low socioeconomic status. Further community-based intervention programs focusing on neighborhood are warranted.

## Supporting information

S1 Table The coefficients at individual, family and block levels and the fraction of total variance due to the variation at the family and block levels. (DOCX)

S1 File Study protocol. (PDF)

S2 File TREND Statement Checklist. (PDF)

S3 File Baseline questionnaire. (PDF)

S4 File Post survey questionnaire. (PDF)

S5 File Focus group interview guide. (PDF)

## References

[1] Schuck AM, Rosenbaum DP. Promoting Safe and Health Neighborhoods: What Research Tells Us about Intervention2006.

[2] Kawachi I, Berkman L. Social cohesion, social capital, and health. Social Epidemiology. 2000:174–90.

[3] Forrest R, Kearns A. Social cohesion, social capital and the neighbourhood. Urban studies. 2001;38(12):2125–43.

[4] Drukker M, Buka SL, Kaplan C, McKenzie K, Van Os J. Social capital and young adolescents' perceived health in different sociocultural settings. Soc Sci Med. 2005;61(1):185–98. Epub 2005/04/26. doi: 10.1016/j.socscimed.2004.11.041 15847971 10.1016/j.socscimed.2004.11.041

[5] Fujisawa Y, Hamano T, Takegawa S. Social capital and perceived health in Japan: an ecological and multilevel analysis. Soc Sci Med. 2009;69(4):500–5. Epub 2009/07/07. doi: 10.1016/j.socscimed.2009.05.046 19577353 10.1016/j.socscimed.2009.05.046

[6] Sapag JC, Aracena M, Villarroel L, Poblete F, Berrocal C, Hoyos R, et al Social capital and self-rated health in urban low income neighbourhoods in Chile. J Epidemiol Community Health. 2008;62(9):790–2. Epub 2008/08/15. doi: 10.1136/jech.2006.052993 18701728 10.1136/jech.2006.052993

[7] Clark CJ, Guo H, Lunos S, Aggarwal NT, Beck T, Evans DA, et al Neighborhood cohesion is associated with reduced risk of stroke mortality. Stroke. 2011;42(5):1212–7. doi: 10.1161/STROKEAHA.110.609164 21493914 10.1161/STROKEAHA.110.609164 PMC3102433

[8] Nguyen TT, Rist PM, Glymour MM. Are self-reported neighbourhood characteristics associated with onset of functional limitations in older adults with or without memory impairment? J Epidemiol Community Health. 2016 Epub 2016/05/08. doi: 10.1136/jech-2016-207241 27154180 10.1136/jech-2016-207241

[9] Mair C, Roux AVD, Shen M, Shea S, Seeman T, Echeverria S, et al Cross-sectional and longitudinal associations of neighborhood cohesion and stressors with depressive symptoms in the multiethnic study of atherosclerosis. Ann Epidemiol. 2009;19(1):49–57. doi: 10.1016/j.annepidem.2008.10.002 19064189 10.1016/j.annepidem.2008.10.002 PMC2763272

[10] Fisher KJ, Li F, Michael Y, Cleveland M. Neighborhood-level influences on physical activity among older adults: a multilevel analysis. J Aging Phys Act. 2004;12(1):45–63. 15211020 10.1123/japa.12.1.45

[11] Patterson JM, Eberly LE, Ding Y, Hargreaves M. Associations of smoking prevalence with individual and area level social cohesion. J Epidemiol Community Health. 2004;58(8):692–7. Epub 2004/07/15. doi: 10.1136/jech.2003.009167 15252073 10.1136/jech.2003.009167 PMC1732846

[12] Cradock AL, Kawachi I, Colditz GA, Gortmaker SL, Buka SL. Neighborhood social cohesion and youth participation in physical activity in Chicago. Soc Sci Med. 2009;68(3):427–35. Epub 2008/11/28. doi: 10.1016/j.socscimed.2008.10.028 19036490 10.1016/j.socscimed.2008.10.028

[13] Abbott S, Freeth D. Social capital and health starting to make sense of the role of generalized trust and reciprocity. J Health Psychol. 2008;13(7):874–83. doi: 10.1177/1359105308095060 18809638 10.1177/1359105308095060

[14] FAMILY Project Cohort: Baseline Findings Kwun Tong District-specific report. https://familycohort.sph.hku.hk/en/sites/default/files/Kwun%20Tong%20Report%2020150203_0.pdf. . 2015.

[15] Census and Statistic Department, Hong Kong Government. Persons from the Mainland Having Resided in Hong Kong for Less Than 7 Years (PMRs) by District Council district, 2001, 2006 and 2011. http://www.census2011.gov.hk/en/main-table/F312.html.

[16] Steptoe A, Feldman PJ. Neighborhood problems as sources of chronic stress: development of a measure of neighborhood problems, and associations with socioeconomic status and health. Ann Behav Med. 2001;23(3):177–85. 11495218 10.1207/S15324796ABM2303_5

[17] Statistics on Elder Abuse Cases. Hong Kong Social Welfare Department. http://www.swd.gov.hk/en/index/site_pubsvc/page_family/sub_listofserv/id_serabuseelder/. Last updated: November, 2015.

[18] Statistics on child abuse, spouse/cohabitant battering and sex violences cases. Hong Kong Social Welfare Department. http://www.swd.gov.hk/vs/english/stat.html. Last updated: September 2015.

[19] Leung GM, Ni MY, Wong PT, Lee PH, Chan BH, Stewart SM, et al Cohort Profile: FAMILY Cohort. Int J Epidemiol. 2015 Epub 2015/01/27. doi: 10.1093/ije/dyu257 25617647 10.1093/ije/dyu257

[20] Fabrizio CS, Lam TH, Hirschmann MR, Pang I, Yu NX, Wang X, et al Parental emotional management benefits family relationships: A randomized controlled trial in Hong Kong, China. Behav Res Ther. 2015;71:115–24. Epub 2015/06/27. doi: 10.1016/j.brat.2015.05.011 26112397 10.1016/j.brat.2015.05.011

[21] Li WH, Mak YW, Chan SS, Chu AK, Lee EY, Lam TH. Effectiveness of a play-integrated primary one preparatory programme to enhance a smooth transition for children. J Health Psychol. 2013;18(1):10–25. Epub 2012/02/11. doi: 10.1177/1359105311434052 22322991 10.1177/1359105311434052

[22] Yu X, Stewart SM, Chui JP, Ho JL, Li AC, Lam TH. A pilot randomized controlled trial to decrease adaptation difficulties in chinese new immigrants to Hong Kong. Behav Ther. 2014;45(1):137–52. Epub 2014/01/15. doi: 10.1016/j.beth.2013.10.003 24411121 10.1016/j.beth.2013.10.003

[23] Ho HC, Mui M, Wan A, Ng YL, Stewart SM, Yew C, et al Happy Family Kitchen II: A Cluster Randomized Controlled Trial of a Community-Based Family Intervention for Enhancing Family Communication and Well-being in Hong Kong. Front Psychol. 2016;7:638 Epub 2016/05/21. doi: 10.3389/fpsyg.2016.00638 27199864 10.3389/fpsyg.2016.00638 PMC4853398

[24] Stewart SM, Fabrizio CS, Hirschmann MR, Lam TH. Developing community-based preventive interventions in Hong Kong: a description of the first phase of the family project. BMC Public Health. 2012;12:106 Epub 2012/02/09. doi: 10.1186/1471-2458-12-106 22309796 10.1186/1471-2458-12-106 PMC3297497

[25] McLaren L, Hawe P. Ecological perspectives in health research. J Epidemiol Community Health. 2005;59(1):6–14. doi: 10.1136/jech.2003.018044 15598720 10.1136/jech.2003.018044 PMC1763359

[26] Sallis JF, Owen N, Fisher EB. Ecological models of health behavior. Health behavior and health education: Theory, research, and practice. 2008;4:465–86.

[27] Lamb P. The Learning Family: a NIACE briefing note. http://shop.niace.org.uk/media/catalog/product/L/e/Learning-Family-briefing.pdf. 2009.

[28] Chan SS, Viswanath K, Au DW, Ma CM, Lam WW, Fielding R, et al Hong Kong Chinese community leaders' perspectives on family health, happiness and harmony: a qualitative study. Health Educ Res. 2011;26(4):664–74. Epub 2011/05/04. doi: 10.1093/her/cyr026 21536713 10.1093/her/cyr026

[29] Adams J, Witten K, Conway K. Community development as health promotion: evaluating a complex locality-based project in New Zealand. Community Development Journal. 2009;44(2):140–57.

[30] Speer PW, Jackson CB, Peterson NA. The relationship between social cohesion and empowerment: support and new implications for theory. Health Educ Behav. 2001;28(6):716–32. Epub 2001/11/27. doi: 10.1177/109019810102800605 11720274 10.1177/109019810102800605

[31] Powell E, Woodfield LA, Nevill AM. Increasing physical activity levels in primary school physical education: The SHARP Principles Model. Prev Med Rep. 2016;3:7–13. Epub 2016/02/05. doi: 10.1016/j.pmedr.2015.11.007 26844179 10.1016/j.pmedr.2015.11.007 PMC4733067

[32] Peterson KE, Sorensen G, Pearson M, Hebert JR, Gottlieb BR, McCormick MC. Design of an intervention addressing multiple levels of influence on dietary and activity patterns of low-income, postpartum women. Health Educ Res. 2002;17(5):531–40. Epub 2002/11/01. 12408198 10.1093/her/17.5.531

[33] Fabrizio CS, Stewart SM, Ip AK, Lam TH. Enhancing the parent–child relationship: A Hong Kong community-based randomized controlled trial. Journal of Family Psychology. 2014;28(1):42 doi: 10.1037/a0035275 24364364 10.1037/a0035275

[34] Schafer JL. Multiple imputation: a primer. Stat Methods Med Res. 1999;8(1):3–15. Epub 1999/05/29. doi: 10.1177/096228029900800102 10347857 10.1177/096228029900800102

[35] 2011 Population Census - Fact Sheet for Tsui Ping (North) Estate in Kwun Tong District Council District. http://www.census2011.gov.hk/en/major-housing-estate/10170.html.

[36] 2011 Population Census - Fact Sheet for Shun Tin Estate in Kwun Tong District Council District. http://www.census2011.gov.hk/en/major-housing-estate/10140.html.

[37] Sampson RJ, Raudenbush SW, Earls F. Neighborhoods and violent crime: A multilevel study of collective efficacy. Science. 1997;277(5328):918–24. 9252316 10.1126/science.277.5328.918

[38] Cronbach LJ. Coefficient alpha and the internal structure of tests. Psychometrika. 1951;16(3):297–334.

[39] Cohen J. Statistical power analysis for the behavioral sciences (revised ed.). New York: Academic Press; 1977.

[40] Braun V, Clarke V. Using thematic analysis in psychology. Qualitative Research in Psychology. 2006;3(2):77–101.

[41] Golden SD, Earp JA. Social ecological approaches to individuals and their contexts: twenty years of health education & behavior health promotion interventions. Health Educ Behav. 2012;39(3):364–72. Epub 2012/01/24. doi: 10.1177/1090198111418634 22267868 10.1177/1090198111418634

[42] Chen G-M, Chen G, Ma R. The impact of harmony on Chinese conflict management. Chinese conflict management and resolution. 2002:3–17.

