# Supplementary material for: A community based intervention program to enhance neighborhood cohesion: The Learning Families Project in Hong Kong
Source: PLoS One. 2017 Aug 21;12(8):e0182722. doi: 10.1371/journal.pone.0182722 (PMC5565197; doi:10.1371/journal.pone.0182722)
Supplement: S1 Table — (DOCX) [file pone.0182722.s001.docx]

Supplementary Table. The coefficients at individual, family and block levels and the fraction of total variance due to the variation at the family and block levels

|  |  | Overall neighborhood cohesion scale^a^ | Item 1  People around here are willing to help their neighbors | Item 2  This is a close-knit neighborhood | Item 3  People in this neighborhood can be trusted | Item 4  People in this neighborhood generally do not get along with each other | Item 5  People in this neighborhood do not share the same values |
| --- | --- | --- | --- | --- | --- | --- | --- |
| Estate | Control | 0 | 0 | 0 | 0 | 0 | 0 |
|  | Intervention | 0.05 | 0.10 | 0.13* | 0.14* | -0.08 | -0.08 |
| Age | <18 | 0 | 0 | 0 | 0 | 0 | 0 |
|  | 18-44 | 0.28 | 0.28 | 0.09 | 0.14 | 0.33 | 0.56* |
|  | 45-64 | 0.28 | 0.18 | -0.02 | 0.08 | 0.42 | 0.71** |
|  | 65+ | 0.30 | 0.22 | -0.02 | 0.13 | 0.49 | 0.72** |
| Education | No formal education | 0 | 0 | 0 | 0 | 0 | 0 |
| level | Primary | 0.05 | 0.05 | 0.05 | 0.02 | 0.12 | 0.04 |
|  | Secondary or above | -0.05 | -0.01 | -0.07 | -0.01 | -0.03 | -0.08 |
| Random | Family level | 0 | 0.13 | 0 | 0 | 0 | 0 |
| effect | ICC | 0 | 0.02 | 0 | 0 | 0 | 0 |
|  | Block level | 0 | 0 | 0 | 0 | 0.05 | 0 |
|  | ICC | 0 | 0 | 0 | 0 | 0.01 | 0 |

ICC: Intra-cluster correlation coefficient, which is the fraction of total variance due to the variation between clusters

a: Scores in the neighborhood cohesion scale were the average score of items (Items 4 and 5 were reverse coded)

*: Statistically significant at P<0.05

**: Statistically significant at P<0.01
