# Supplementary material for: A community based intervention program to enhance neighborhood cohesion: The Learning Families Project in Hong Kong
Source: PLoS One. 2017 Aug 21;12(8):e0182722. doi: 10.1371/journal.pone.0182722 (PMC5565197; doi:10.1371/journal.pone.0182722)
Supplement: S1 File — (PDF) [file pone.0182722.s002.pdf]

STUDY PROTOCOL

Project Title

Promoting family health, happiness, and harmony through a community-based "Learning Family" campaign

Principal Investigator

Professor Sophia Siu Chee CHAN,
Professor, School of Nursing, The University of Hong Kong

Co-Investigator

Professor Tai Hing LAM,
Chair Professor and Head, Department of Community Medicine, School of Public Health,
The University of Hong Kong

Dr. Catalina Sau Man NG,
Post-doctoral Fellow, School of Nursing, The University of Hong Kong

Study Sites

Tsui Ping (South) and Shun Tin Estates, Kwun Tong district.

Aims of the Project

This study aims to evaluate the effectiveness of a community-based 'Learning Family' campaign to families in Kwun Tong to promote family health, happiness, and harmony (3Hs). The hypothesis of this project is this special designed community-based intervention will lead to a significant increase in 3Hs in the intervention group than the control group. The specific objectives of the study are:

1. To promote family 3Hs among families living in public housing estates at Kwun Tong district through cultivating a cooperative and self-regulated family learning culture.
2. To utilize community resources by building capacity among public housing estate resident leaders

(Estate Management Advisory Committee & Mutual Aid Committee) through training programmes on the concepts of family learning and 3Hs

3. To establish a platform enhancing social networking and neighbourhood support by multiple family-based activities for public housing estate residents organized by resident leaders and social workers.
4. To evaluate various components of the project in terms of its structure, process, and outcomes.
5. To evaluate the impact of training workshop on knowledge and attitudes about the key message of self-regulated learning behaviors and family learning concept.

Outcomes

The primary outcome of the study includes:

(i) Family: Increase of family health, happiness, and harmony (3Hs) measured directly by an increase in 3Hs respective scores.

The secondary outcomes include:

A. Campaign Level

(i) Family: Increase in social capital, family resilience, and neighbourhood cohesion of the family after the whole campaign evaluated by the post-campaign survey.

(ii) Individual: Increase in life satisfaction and marital satisfaction after the whole campaign by the post-campaign survey.

B. Program Level

(i) Improved knowledge and attitudes of the key message of self-regulated learning behaviours, and family learning concepts by program evaluation during several time points of the program.

Estimated Duration and Commencement Data

Proposed start date: November 2010

Proposed study completion date: March 2012

Expected final report date: April 2012

Study Rationale

According to the Social Welfare Department statistics, Kwun Tong has been ranked as the highest for the reported cases of elderly abuse (12.3%), the second and fourth highest for the reported cases of battered spouse (9.2%) and child abuse (7.9%) respectively [1]. To strengthen family well-being in the Kwun Tong community, we adopt a community-based participatory (CBP) approach [2-5] and implement a community-based "Learning Family" campaign in Kwun Tong district with our collaborator, the Christian Family Service Centre (CFSC). The campaign aims to promote family health, happiness, and harmony (3Hs) through cultivating cooperative and self-regulated family learning culture in two public housing estates, Tsui Ping South Estate (intervention) and Shun Tin Estate (control), in Kwun Tong district.

Based on the Social Ecological Model, it is believed that people can change their behavior by different level of effects including individual, interpersonal, community and societal level. It is used as a guiding framework to promote 3Hs through observational learning and participation in community activities. Given that CFSC is the leading non-government organization based in Kwun Tong district, we believe that the proposed campaign will deliver the family 3Hs messages effectively in the community via engaging major stakeholders such as the Estate Management Advisory Committees (EMACs) and the Mutual Aid Committees (MACs).

Study Design

This is a Community-based Participatory Research (CBPR) using both quantitative (cross-sectional and longitudinal surveys) and qualitative (focus groups) methodologies. A two group comparison experimental design is also employed with an intervention housing estate and control housing estate. Process and outcome evaluation will be conducted at different time points throughout the different phases of the project.

The project will be conducted in five phases:

(1) **Phase I**: includes providing training sessions to EMACs & MACs to engage and equip them with the concept of 3Hs and organizing skills to enhance their motivation of participation;

(2) **Phases II:** includes a baseline (1st) survey of the residents in the intervention and control estates, and

a kick-off ceremony of the campaign in Tsui Ping South Estate (intervention estate).

(3) **Phase III:** includes a community-based campaign (1. Promotional activities 2. Training activities 3. Learning activities) for families in the intervention estate to enhance their knowledge and attitudes about the key messages of self-regulated learning behaviours and family learning concepts, and to promote family 3Hs.

(4) **Phase IV:** includes a final (2nd) survey on the intervention and control estates.

(5) **Phase V:** includes an award ceremony and conducting focus groups to explore volunteers', staff's and family members' experiences, thoughts and feelings about the campaign.

Selection of subjects

Residents living in Tsui Ping South Estate (Intervention) and Shun Tin Estate (Control) in Kwun Tong district, including representatives of Estate Management Advisory Committees (EMACs) and Mutual Aid Committees (MACs) will be invited to participate in the study.

Inclusion criteria:

(1) Participants are Hong Kong residents, and are currently residing in the designated estates (Tsui Ping South Estate and Shun Tin Estate) in Kwun Tong district; and
(2) Participants are able to communicate in Cantonese/Putonghua.

Exclusion criteria:

(1) Participants who do not live in Tsui Ping South Estate and Shun Tin Estate, Kwun Tong will be excluded.

Number of participants to be recruited

1. Needs Assessment
   A total 16 of EMAC and MAC members will be recruited to participate in 2 focus group interviews, and they also are invited to participate in the training programme.

2. The EMAC Train-the-Trainers Programme
   About 32 participants who are either EMACs, peer counsellors, or activity instructors from Tsui Ping South Estate or Shun Tin Estate will be recruited to participate in the training programme.

3. Baseline and Final Surveys

Families and residents of two estates will be invited to complete a self-administered survey at baseline and upon completion of the campaign. The sample size required will be 1,300 participants from each of Tsui Ping South Estate and Shun Tin Estate, Kwun Tong. .

4. The Learning Family Campaign

Training Activities: Around of 430 families in intervention estate will be recruited for a special design training programme;

Promotional Activities & Learning Activities: CFSC and the EMACs will conduct 27 community activities with themes in learning family and 3Hs and it is proposed to recruit around 500 families in attendance.

5. Award Ceremony

It is expected that about 500 participants including EMACs, peer counsellors, activity instructors and participating families will attend the award ceremony.

Consent

Participation in the study is totally voluntary. Participants will be required to fill in a consent form before completing the questionnaire or attending the focus group interview. Participants will be provided with a contact telephone number in case they have any query about the project, and they have the right to withdraw from the study at any time points without any consequences.

Procedures

Phase I: Needs Assessment & Train-the-trainers Programme for EMACs and MACs

(i) Pre-campaign Need Assessment Focus Group Interviews –

Prior to the development of the campaign, a needs assessment will be conducted to explore EMACs, MACs' views on family 3Hs, learning needs, resources, and feasibility of the 'Learning Family' campaign at different phases. The information collected will contribute to the development of a practical train-the-trainers program. EMAC and MAC members will be invited to participate in 2 focus groups interviews. The focus group interviews will be conducted by experienced facilitators in both The University of Hong Kong and CFSC. Prior to the focus group interviews, all the participants will first be welcomed by the facilitators, explained the study objectives, and be informed that the whole process will be audio-taped. One of the facilitators will take field notes during the entire process. All the participants' personnel information will be assured to be kept confidential, use only for research purpose, and their participation is

completely voluntary. After obtaining written consent, the participants will first complete a simple questionnaire on their demographic profile. Then, the facilitators will guide the discussion according to the interview guideline and probe for further information where necessary. For each focus group, one of the facilitators will be a CFSC social worker who will lead the discussion. Other facilitators will be members of the research team who will assist the facilitator, audio-tape the discussion and take field notes. Each interview is estimated to last for 50-90 minutes.

(ii) Pre-campaign Train-the-trainers Programme –

The Train-the-trainers programme is specially designed for the EMACs, MACs, peer counsellors, and activity instructors. Prior to the training, participants will complete a pre-assessment questionnaire. Immediately after the training, the participants also will complete a post-assessment questionnaire. The pre- and post- assessment will be used to assess participants' perceptions of the training and their understanding of the knowledge delivered in the training program. Outcome measures such as self-reported competence before and after training will be assessed. A booster session will be held about 6 weeks after the training programme, to build up a horizontal network among volunteers, provide mutual support and training opportunities for volunteers to learn from others. Participants in the booster session will complete a questionnaire to explore their perceptions on the motivators and possible difficulties in motivating residents to join the campaign.

Phase II: Baseline Survey & Kick-off ceremony

A two group experimental design will be used, with two public housing estates in Kwun Tong district as the primary sampling units. The public housing estate- Tsui Ping (South) Estate will be assigned as intervention estate (i.e., exposed to the campaign), whereas Shun Tin Estate as control estate (i.e. not exposed to the campaign).

(i) Questionnaire:

Both intervention and control estates are required to complete a self-administrated pre-survey. It aims to assess the community views on family 3Hs & social capital in a recruitment of 1300 residents from each estate. The questionnaire will include the following measures:

- Roles and responsibilities in family
- Family communication
- Social support
- Satisfaction with the support network
- Neighbourhood cohesion

- Marital satisfaction
- Life satisfaction
- Community participation and resources
- Family resilience

For the Kick-off ceremony, a post-program questionnaire will be used to access satisfaction towards the program, program effectiveness in enhancing family 3Hs and the current status of 3Hs of the residents.

Phase III: Community-based Learning Family Campaign

Intervention group:

(i) Attending the Learning Family Campaign

The CFSC will provide a series of learning family interventions to families in Tsui Ping South Estate. (a) Promotion program will be used to instil the messages of family 3Hs and "learning family" concepts to the residents, promoting the future training workshops and learning family activities to the participants. Program evaluation will be completed by the participant to explore the impact of the program. (b) Training programs aiming to promote learning family and 3Hs concepts to the residents will be implemented. Pre and Post-program evaluation and questionnaire will be used to evaluate the impact of the training workshop regarding the knowledge, self-efficacy and intention to act and attitudes changes in the key message of self-regulated learning behaviours and family learning concepts. (c) Learning programs organized by EMACs will then be followed in order to promote more learning behaviours and the concepts of family 3Hs. Program evaluations will be completed to further evaluate the program impacts.

(ii) At 6 weeks

Those 430 participating families will participate in a booster session and to complete follow up questionnaires at 6 weeks after attending the learning family intervention in order to track their changes and any barriers in actions taken.

Phase IV: Post Survey

Around 1300 residents in both 2 estates residents will be followed up by completing a self-administered post-survey at 3 months after the campaign in order to measure the study outcomes and compared the difference within the residents in 2 estates for further measure the actual impact of campaign.

Phase V: Award Ceremony and Post-campaign Focus Group Interviews

(a) Post-Assessment Survey

A post-assessment survey of 500 people will be completed immediately after by attending the award ceremony to assess the outcomes of gain in knowledge, attitude change and satisfaction. Number of residents from various estates attending the ceremony and number of participants willing to provide contact information will be measured.

(b) Focus group interviews

After the completion of campaign and award ceremony, focus group interviews for EMACs workers will be conducted to explore their experiences with the campaign including perceptions of challenges, collective and personal growth, and experiences sharing with others. Focus group interviews for participating families will also be conducted to examine family members' experiences, thoughts and feelings on the campaign.

Instruments

I. Focus group interviews

An interview guideline was developed to explore EMACs and workers' experiences with the campaign including perceptions of challenges, personal growth and sharing of experiences with others. Besides, another interview guideline was also developed to explore participating families' experiences, thoughts and feelings with the campaign.

II. Questionnaire surveys

A number of questionnaires will be developed and adapted from previous studies of the investigators, other worldwide validated measures, and the pilot study. These include:

(1) Baseline and final questionnaires for families from both estates to measure the primary and secondary outcomes in the community views on family 3Hs & social capitals;

(2) Pre and Post-assessment questionnaires after the learning family training programme to evaluate the impact of training programme on knowledge and attitudes changes in the key messages of self-regulated learning behaviours and family learning concepts;

(3) Post-assessment questionnaires after the learning family promotion and learning programmes for further evaluate the programs impact.

<u>Methods of Statistical Analysis</u>

(a) Focus group interviews

All qualitative data will be tape recorded and transcribed verbatim. Transcripts will be then analyzed and coded using a thematic content analysis [6]. Three coding phases will be involved:

(1) open coding will be performed whereby each segment of the transcribed interviews will be coded using a paraphrase that connoting these words;

(2) axial coding will then proceed with clustering the descriptive codes into groups of related subcategories; and

(3) these categories will be refined and grouped around common themes using selective coding [7].

(b) Questionnaire surveys

All statistical analyses will be conducted using STATA version 13.0. Descriptive statistics such as frequencies, percentages, and means will be calculated for demographic characteristics. Chi-square tests and t-tests will be used to detect a significant difference between pre- and post-training scores of outcome variables. Regression models will be performed to evaluate the effectiveness of the intervention and identify factors associated with family health, happiness, and harmony (3Hs).

Safety Parameters

No drug investigation, unusual or discomforting procedures will be used in this study and no hazards associated will be raised with the study investigation.

Direct Access to Source Data/Documents

The study data will be stored in the CD-ROM and locked in a filing cabinet with keys kept by the principal investigator. Only the investigators and researchers of the project will be permitted to access to raw data and/or study record. The data will be kept for 10 years or longer after the study is completed. Individual participants will not be directly identifiable from the dataset to be used for analysis.

Dissemination of Study Result

The research findings will be reported to The Hong Kong Jockey Club Charities Trust for project evaluation, disseminated in local and international symposium and conferences, as well as published in international peer reviewed journals.

Conflict of Interest

None

Financing and Insurance

This project is sponsored by The Hong Kong Jockey Club Charities Trust.

References

1. District Statistic, Social Welfare Department, HKSAR. 2010

2. Berge, J.M., T.J. Mendenhall, and W.J. Doherty, *Using community-based participatory research (CBPR) to target health disparities in families.* Family Relations, 2009. **58**(4): p. 475-488.

3. Macaulay, A.C., *Promoting Participatory Research by Family Physicians*, in *Annals of Family Medicine*2007, Annals of Family Medicine. p. 557-560.

4. Shalowitz, M.U., et al., *Community-based participatory research: A review of the literature with strategies for community engagement.* Journal of Developmental and Behavioral Pediatrics, 2009. **30**(4): p. 350-361.

5. White, G.W., M. Suchowierska, and M. Campbell, *Developing and systematically implementing participatory action research.* Archives of Physical Medicine and Rehabilitation, 2004. **85**(Supplement 2): p. 3-12.

6. Corbin, J. and A. Strauss, *Basics of qualitative research*2008, Los Angeles, CA: Sage Publications.

7. Krippendorff, K., *Content analysis: An introduction to its methodology* 2004, Thousand Oaks, CA: Sage Publications.
