# Supplementary material for: A community based intervention program to enhance neighborhood cohesion: The Learning Families Project in Hong Kong
Source: PLoS One. 2017 Aug 21;12(8):e0182722. doi: 10.1371/journal.pone.0182722 (PMC5565197; doi:10.1371/journal.pone.0182722)
Supplement: S4 File — (PDF) [file pone.0182722.s005.pdf]

Name in Chinese:___________________ Contact number: ___________________________

Learning Family Project
Follow-up Survey

Date of questionnaire completion: ____Year/____Month/____Day

Please answer **all questions** of each part, the information you provided will be kept **strictly confidential**, and will be used for research analysis of this project only.

Note: Please clearly put a " ✔ " on the selected circle. For example: ①✓ ② ③

1. From which of the following source you get this questionnaire?

①Mail box ②Mutual Aid Committee ③Block counter ④ Housing Authority ⑤Activity of Learning Family Project ⑥Other, please specify: _______________

Part 1: Family Harmony

1 In general, I get along well with my family.	①Strongly agree	②Agree	③Neutral/ No comments	④Disagree	⑤Strongly disagree

2 Do you think **your family is harmonious**?

(Please circle a number to indicate your level of family **harmony**, **0 is NOT AT ALL** HARMONIOUS, **10 is VERY** HARMONIOUS)

0 1 2 3 4 5 6 7 8 9 10

NOT AT ALL HARMONIOUS ◄———— Average ————► **VERY** HARMONIOUS

Part 2: Family Role

1 Are you satisfied or dissatisfied with the balance between your job and home life? ① Yes ② No

2. Number of family roles for which you were mostly or fully responsible, including: earning money to support the family; taking care of children; taking care of another household (for example: (maternal) grandparents)

① 1 ② 2 ③ 3 ④ 4 or above

Part 3: Family Communication

1 In **the past 7 days**, how long have you spent on average **per day** to communicate or chat with your family?

[ ] Hour [ ] Minute (fill in a number)

2 Do you think you have enough communication time with your family?

①Extremely enough ② Enough ③ Average ④ Not enough ⑤ Extremely not enough

Part 4: Interpersonal Relationships and Support

1 How many friends do you feel at ease with, can talk to about private matters, or can call on for help?

[ ] (fill in a number)

2 How many family members do you feel at ease with, can talk to about private matters, or can call on for help?

[ ] (fill in a number)

Part 5: Social Support

1 When you were sick, need help with household task, financial support, or get to the doctor, do you find it easy to seek for the help from family or friends?

① Very difficult ② Difficult ③ Average ④ Easy ⑤ Very Easy

2 How many people you are in regular contact (including family, relatives, friends & neighbours)?

[ ] (fill in a number)

In the **past ONE month**, how frequent you were in regular contact with the following persons?

	At least once per day	At least once a week	At least once a month	Never
3 family members:	①	②	③	④
4 relatives:	①	②	③	④
5 friends:	①	②	③	④
6 neighbours:	①	②	③	④

Are you satisfied with…	Very dissatisfied	Dissatisfied	Average	Satisfied	Very satisfied
7 support received from family members?	①	②	③	④	⑤
8 support received from your relatives?	①	②	③	④	⑤
9 support received from your friends?	①	②	③	④	⑤
10 support received from your neighbours?	①	②	③	④	⑤

Part 6: Neighbourhood Cohesion

	Strongly agree	Agree	Neutral	Disagree	Strongly disagree
1 People around here are willing to help their neighbours.	①	②	③	④	⑤
2 This is a close-knit neighbourhood.	①	②	③	④	⑤
3 People in this neighbourhood can be trusted.	①	②	③	④	⑤
4 People in this neighbourhood generally do not get along with each other.	①	②	③	④	⑤
5 People in this neighbourhood do not share the same values.	①	②	③	④	⑤

Part 7: Life Satisfaction Scale

	Strongly disagree	Disagree	Neutral	Agree	Strongly agree
	①	②	③	④	⑤
	①	②	③	④	⑤

	①	②	③	④	⑤
1 In most ways my life is close to my ideal.	①	②	③	④	⑤
2 The conditions of my life are excellent.					
3 I am satisfied with my life.					
4 So far I have gotten the important things I want in life.					

5 During the past half year, have you thought of quitting your job? ① Yes ② No ③ Not applicable

6 Marital status: ① Single ② Married ③ Cohabitation ④ Divorced ⑤ Separated ⑥ Widowed

**If you are <u>married</u>, please answer the following two questions:**	Strongly disagree	Disagree	Neutral	Agree	Strongly agree
6.1 Our relationship is a perfect success.	①	②	③	④	⑤
6.2 I am very happy with how we handle role responsibilities in our marriage.	①	②	③	④	⑤

Part 8: Level of Happiness

1 All things considered, would you say you are: ① Very Happy ② Happy ③ Not very happy ④ Not happy at all

2 Do you think **your family is happy**?

(Please circle a number to indicate your level of family **happiness**, **0 is NOT** HAPPY, **10 is VERY** HAPPY)

0 1 2 3 4 5 6 7 8 9 10

NOT HAPPY ← Average → **VERY** HAPPY

Part 9: Level of Health

1 In general, you think your health status is: ①Excellent ②Very good ③Good ④Fair ⑤ Bad

2 In physical health aspect:

① Strongly unsatisfactory ② Very unsatisfactory ③ Unsatisfactory ④ Average ⑤ Satisfactory ⑥ Very satisfactory ⑦ Strongly satisfactory

3 In mental and psychological health aspect:

① Strongly unsatisfactory ② Very unsatisfactory ③ Unsatisfactory ④ Average ⑤ Satisfactory ⑥ Very satisfactory ⑦ Strongly satisfactory

4 Do you think **your family is healthy**?

(Please circle a number to indicate your level of family **health**, **0 is NOT** HEALTHY, **10 is VERY** HEALTHY)

0 1 2 3 4 5 6 7 8 9 10

NOT HEALTHY ← Average → **VERY** HEALTHY

5. Do you smoke cigarettes?

① No, I have never tried to smoke (↳**go to q8**)　② No, I have quitted smoking for 6 months or below　③ No, I have quitted smoking for more than 6 months

④ Yes, I smoke daily　⑤ Yes, I smoke occasionally

If you are a smoker, please fill in the following three questions:

6. Are you thinking of quitting smoking?　①Yes (↳**continue with q7**)　②No (↳**go to q8**)

7. When do you want to quit smoking? (choose 1 only)

① I have already stopped　② I will stop smoking today　③ I will stop within the next 7 days

④ I will stop in the next 30 days　⑤ I will stop in the next 6 months　⑥ I will stop after 6 months or later

8. How frequent do you drink alcohol (e.g. 1 can of beer, 1 glass of house wine or 1 peg of liquor)?

①Never　②Drink less than once a month　③ Drink 1 – 3 days **a month**　④ Drink 1 – 3 days **a week**

⑤ Drink 4 – 6 days a week　⑥ Drink daily　⑦ Quitted　⑧ Only drink on special occasions

Part 10: Power, Participation and Resources in Community

	Strongly disagree	Disagree	Neutral	Agree	Strongly agree
1 I can influence the decisions that this community makes.	①	②	③	④	⑤
2 By working together, people in my community can influence decisions that affect the community.	①	②	③	④	⑤
3 People in my community work together to influence decisions on the societal level.	①	②	③	④	⑤
4 I am satisfied with the amount of influence I have over decisions that affect my community.	①	②	③	④	⑤

5 Have you participated in the activities held in your estate?　① Seldom　② Occasionally　③ Sometimes　④ Most of the time　⑤ Always

6 Do you have volunteer work?　① Yes　② No

7 Do you think the resources in your community are enough (such as transport facilities, recreation facility, shopping and leisure, etc.)?　①Enough　②Not enough

8 Have you used the service provided by the social service organization in your community?　① Yes　② No

Part 11: Family Resilience Scale

My family and I think that…	Strongly agree	Agree	Slightly agree	Neutral	Slightly disagree	Disagree	Strongly disagree
1 We all have input into major family decisions.	①	②	③	④	⑤	⑥	⑦
2 We can work through difficulties as a family.	①	②	③	④	⑤	⑥	⑦
3 We feel free to express our opinions.	①	②	③	④	⑤	⑥	⑦
4 We share responsibility in the family.	①	②	③	④	⑤	⑥	⑦
5 We ask neighbours for help and assistance.	①	②	③	④	⑤	⑥	⑦
6 We can depend upon people in this community.	①	②	③	④	⑤	⑥	⑦
7 We know there is community help if there is trouble.	①	②	③	④	⑤	⑥	⑦
8 We know we are important to our friends.	①	②	③	④	⑤	⑥	⑦
9 We feel we are strong in facing big problems.	①	②	③	④	⑤	⑥	⑦

Part 12: Family 3Hs – Health, Happiness and Harmony

In the past 7 days, have you actively done the following(s) to increase the 3Hs (Health, Happiness and Harmony) in your family? (You can choose more than one)

	Yes	No		Yes	No
a. Say thank you to family members	○	○	g. Praise family members	○	○
b. Enjoy fresh fruits & vegetables with family	○	○	h. Enjoy food with low fat, low sodium, low sugar and high fiber (“3 low 1 high” rule) with your family	○	○
c. Chat with family	○	○	i. Share happy experience with your family	○	○
d. Take time to walk or do exercise with family	○	○	j. Eat slowly with family	○	○
e. Criticize family members less often	○	○	k. Help to cook/ prepare/ clear /wash dishes	○	○
f. Encourage family members to be optimistic when facing unhappy incidents	○	○	l. Other (Please clarify):		

Part 13：Overall

**In the past one year**, have you?	Yes	No
1 participated in the activities of **FAMILY: A Jockey Club Initiative for a Harmonious Society**?	①	②
2 participated in the activities of **Learning Family Project**?	①	②
3 implemented the suggestions from the “Learning Family Project?	①	②
4 used the Learning Family Booklet distributed during activities？	①	②
5 learnt together with family members (E.g. outdoor activities, competitions, environment education, family life education, interest class, language course)?	①	②

6 Are you intend to start learning with family member?
①next 1-3 months ② next 4-6 months ③No intention to start

Part 14: Personal Background Information

1 Gender: ①Male ②Female

2 Age:_________ (please specify your age, or choose from the below answer)

①Below 18 ②18-24 ③25-34 ④35-44
⑤45-54 ⑥55-59 ⑦60-64 ⑧65 or above

3 Religious belief: ① Christianity ② Catholic ③Buddhism ④Taoism ⑤Others:__________ ⑥None

4 Where were you born? ①Hong Kong ② Guangdong Province ③Other Province in China ④Others: ___________________

5 How long have you lived in Hong Kong? ①1 year or less ②2-3 years ③ 4-6 years ④7 years or above

6 How many family members are living with you (excluding yourself & domestic helper)? [ ] (fill in a number)

7 Who is living with you? ①Spouse ②Children ③Father ④Mother ⑤Siblings ⑥Grandfather/mother ⑦Maternal grandfather/mother ⑧Grand children ⑨None ⑩Other

8 How many children under 18 do you have? ①None ②1 ③2 ④3 ⑤4 ⑥5 or above

9 How many family members are dependent on you for support (excluding yourself)? ①None ②1 ③2 ④3 ⑤4 ⑥5 ⑦6 or above

10 Your education level: ① No formal education ②Primary ③ Secondary 1-3 ④ Secondary 4-5 ⑤ Matriculated (Secondary 6-7) ⑥ Non-degree tertiary ⑦ Degree tertiary or above

11 Your working status: ①Student ② Self-employed ③Employed ④ Job-waiting/ Unemployed ⑤ Housewife ⑥Retired

12 Your family income (monthly): ①Less than$4,000 ②$4,000-$7,999 ③$8,000-$9,999 ④$10,000-$14,999 ⑤$15,000-$19,999 ⑥$20,000-$29,999 ⑦$30,000 or above

13 You think your family financial status is: ① Enough with surplus ② Just enough ③Need to reduce expenditure ④ Serious deficit

End of the questionnaire! Thank you for your participation!
