# Supplementary material for: A community based intervention program to enhance neighborhood cohesion: The Learning Families Project in Hong Kong
Source: PLoS One. 2017 Aug 21;12(8):e0182722. doi: 10.1371/journal.pone.0182722 (PMC5565197; doi:10.1371/journal.pone.0182722)
Supplement: S5 File — (PDF) [file pone.0182722.s006.pdf]

Interview guide for family based focus groups for "Learning Family" Project

I. Process Evaluation

1. Let's recall what you have learnt from "Learning Family" Project. (training, learning, booster)
2. How do you think of this programme? Which part of the programme impressed you the most? What have you felt and experienced in this programme?
3. For those who did not attend the programme, were there any reasons for not attending this event?

II. Outcome Evaluation

1. What have you gained from this programme? Is there anything you have learnt? Please explain with examples. (After your family members participated in this programme, do you think they have also learnt something from it? Please explain with examples.)
2. The main theme of this programme is "Learning Family". How do you interpret this concept at this moment? [Hint: "Learning Family" refers to a family improves the relationship between each family member through the process of learning together.]
3. After participating in the programme, have you tried to lead your family members to learn something or complete certain tasks together so as to improve family relationship? If yes, what have you done with your family members? How was the result? Have you faced any difficulties? How did you overcome the challenges? (After your family members participated in the programme, have they actively led the whole family to learn something or complete certain tasks together so as to improve family relationship? If yes, please explain with examples. How do you feel about their performance?)
4. If you/your family members could not manage to lead the whole family to learn something or complete certain tasks together after the programme, what were the possible reasons for it? Do you think you/your family members have the ability to overcome the difficulties faced? If no, why not?
5. What is the influence of this programme on your family? What have been improved or changed? Please explain with examples. [Hint: The relationship between family members, family happiness, health and harmony] If there have been no improvements or changes, please explain.
6. "Learning Family" Project has been held in the district in the past 6 months, have you noticed any changes in the district? How do you think of these changes? Please explain with examples. Have you noticed any changes in the relationships among neighbours? If yes, please explain with examples.

III. Opinions/suggestions for the project

1. What kind of roles do the Estate Management Advisory Committee (EMAC) and Mutual Aid Committees (MAC) play? What have they done and how do you think of their performance? Are there any areas for improvement?
2. How do you think of the approach of holding promotional activities in the district through the Estate Management Advisory Committee (EMAC) and Mutual Aid Committees (MAC)?
3. How do you think if we keep on promoting any programmes like "Learning Family"? Do you have any suggestions for improvement?
4. Do you have any preference for the kind of activities that could be organized in the district so as to improve family happiness, health and harmony as well as the relationship among neighbours?
